# The Importance of Small Extracellular Vesicles in the Cerebral Metastatic Process

**DOI:** 10.3390/ijms23031449

**Published:** 2022-01-27

**Authors:** Flaviu Tămaș, Rodica Bălașa, Doina Manu, Gabriel Gyorki, Rareș Chinezu, Corina Tămaș, Adrian Bălașa

**Affiliations:** 1 Doctoral School of University of Medicine, Pharmacy, Science and Technology “George Emil Palade”, 540142 Târgu Mureș, Romania; flaviu_tamas1989@yahoo.com (F.T.); rodica.balasa@umfst.ro (R.B.); rchinezu@yahoo.com (R.C.); adrian.balasa@yahoo.fr (A.B.); 2 Department of Neurosurgery, Emergency Clinical County Hospital, 540136 Târgu Mureș, Romania; gabigyorki@yahoo.com; 3 Department of Neurology, Emergency Clinical County Hospital, 540136 Târgu Mureș, Romania; 4 Center for Advanced Pharmaceutical and Medical Research, 540139 Târgu Mures, Romania; doinaramonamanu@gmail.com

**Keywords:** brain, small extracellular vesicles, metastases, exosomes, oncoproteins, cancer

## Abstract

Brain metastases represent more than 50% of all cerebral tumors encountered in clinical practice. Recently, there has been increased interest in the study of extracellular vesicles, and the knowledge about exosomes is constantly expanding. Exosomes are drivers for organotropic metastatic spread, playing important roles in the brain metastatic process by increasing the permeability of the blood–brain barrier and preparing the premetastatic niche. The promising results of the latest experimental studies raise the possibility of one day using exosomes for liquid biopsies or as drug carriers, contributing to early diagnosis and improving the efficacy of chemotherapy in patients with brain metastases. In this review, we attempted to summarize the latest knowledge about the role of exosomes in the brain metastatic process and future research directions for the use of exosomes in patients suffering from brain metastatic disease.

## 1. Introduction

Cerebral metastatic disease is one of the most feared complications of cancer, and brain metastasis represents more than 50% of all brain tumors seen clinically. The primary source of cerebral metastatic disease is most often lung cancer followed by breast cancer. Up to 30% of cancer patients will ultimately present with cerebral metastases. Since the central nervous system lacks a classical lymphatic system, hematogenous spread is usually the dissemination mechanism [1].

In the past decade, major advances have been made to our knowledge about extracellular vesicles. Technical difficulties in the isolation and characterization of pure populations of specific subtypes result in the co-isolation of extracellular vesicles of different subcellular origins. Therefore, the term “exosome” that is used in many articles nowadays actually refers to a mixture of small extracellular vesicles (small EVs) containing both exosomal, as well as non-exosomal, components. This is why the recommendation of using the term “small EVs” instead of “exosomes” is preferable, unless the microvesicular body origin of EVs has been clearly established [2,3,4,5,6]. Due to this agreement, we decided to refer to exosomes as small EVs in the following text.

Extracellular vesicles are grouped based on their sizes, which may vary between 20 nm and 1000 nm, and based on the releasing mechanism, being represented by exosomes (small EVs), microvesicles, and apoptotic bodies. 

Recently, interest in studying small EVs has increased. They drive organotropic metastatic spreading by preferentially accumulating at future metastatic sites [7,8]. Organotropism is the organ specificity of metastases dictated by tumor-derived small EVs, which even today raises many unanswered questions [9]. These small, single-membrane extracellular vesicles that are released by all cell lineages in the human body in a healthy or a pathological state play an important role in noncanonical intercellular communication, among other roles [10]. Their lipid bilayer allows them to safely travel within the bloodstream and join with their target cells [11,12]. With diameters ranging from 20 nm to 100 nm and densities varying from 1.1 g/mL to 1.2 g/mL, small EVs transport various proteins; lipids; and genetic material (messenger RNA, microRNA, and DNA). Small EV cargo is crucial to intercellular communication, which can also modify the extracellular matrix, contributing to the metastatic process. They transport functional RNA to other cells and DNA, which, if sequenced, can reveal the DNA of the originating cell [13,14]. 

Cancer cells have been noted for their enhanced secretion of small EVs with altered contents compared with their noncancerous counterparts. Tumor cells release significant amounts of small EVs that promote tumor growth; inhibit the immune system; and maintain a pro-metastatic phenotype by stimulating angiogenesis, invasion, and proliferation in the hosting cells. Studies have confirmed that small EVs released by primary tumor cells create a favorable environment in the secondary organs by manipulating non-immune stromal cells, mesenchymal stem cells, epithelial cells, or fibroblasts, thus preparing the premetastatic niche for the arriving tumoral cells. Additionally, exosomal release is important in driving the anterior–posterior cellular polarity used by fast-migrating cells such as human leucocytes [14,15,16,17,18]. Small EVs contribute to treatment resistance in cancer patients. For example, breast and prostate chemo-resistant cancer cells can release small EVs with high levels of the MDR-1 (multidrug-resistant) protein, which can be transferred into chemo-sensitive cancer cells and confer chemotherapy resistance [19]. 

Small EVs have been isolated from human body fluids, including plasma, cerebrospinal fluid (CSF), urine, amniotic fluid, breast milk, bronchoalveolar lavage, semen, and malignant ascites [15]. Many techniques are used to isolate small EVs, including differential centrifugation, immunoaffinity capture, sucrose or iodixanol gradients, lipid nanoprobes, and isolation using commercial kits [20]. Each method has its advantages and disadvantages, but the purest small EVs samples can be obtained by ultracentrifugation and density gradient purification, although this method offers a decreased yield [21].

## 2. Exosomal Biogenesis

**Figure 1:**
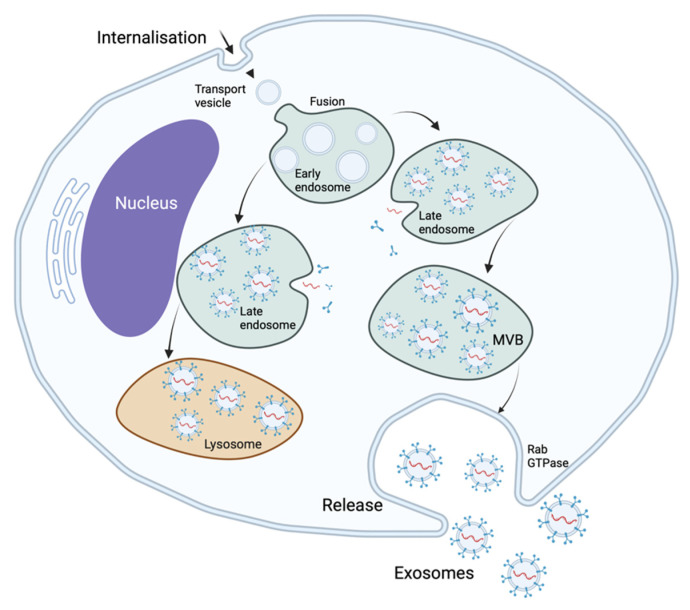
Exosomal biogenesis.

Extracellular vesicles can be classified by their biogenesis mechanisms. Exosomal biogenesis is a mechanism of cellular protein quality control. Exosomes develop from the endosomal system, being formed as intraluminal vesicles within the multivesicular bodies. Invagination of the plasmatic membrane gives rise to early endosomes that, after fusing with endocytic vesicles, become recycling endosomes and late endosomes. Inward budding of the endosomal membrane results in the accumulation of intraluminal vesicles within the late endosomes. These late endosomes containing intraluminal vesicles are termed “multivesicular bodies” and may fuse with lysosomes or with the cellular membrane. If the multivesicular bodies fuse with lysosomes, their content is degraded, but if they fuse with the cellular membrane, they release intraluminal vesicles into the extracellular space as exosomes—Figure 1 [14,22].

**Figure 2:**
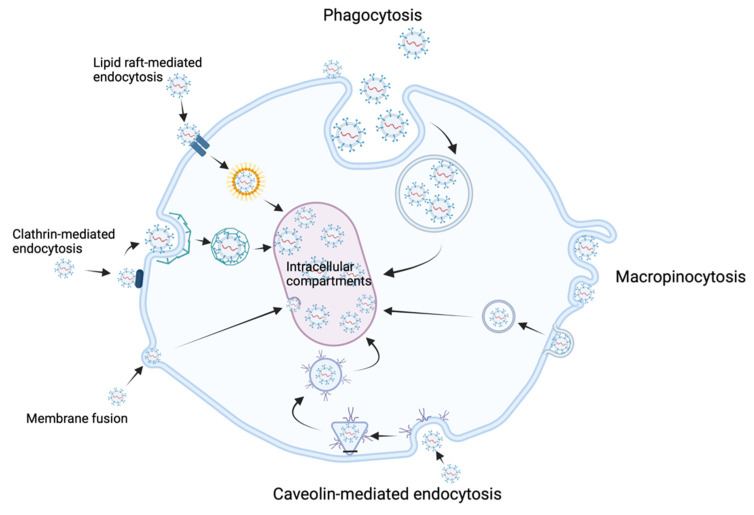
Exosomal uptake mechanisms.

Exosomal biogenesis is catalyzed by many factors, including tetraspanins such as CD9 and CD63 that accumulate in the endosomal membrane, reorganizing it. These tetraspanins can be used as “specific” exosomal biomarkers along with CD81, another tetraspanin specific to exosomal biogenesis. The endosomal sorting complexes required for transport (ESCRT) play an important role in the formation of intraluminal vesicles. Four different ESCRT types—O, I, II, and III—have been identified so far [3,21]. Exosomal release is facilitated by members of the Rab GTPase protein family, such as Rab 7, which has been shown to facilitate exosomal secretion in breast cancer cells [18]. Exosomal release is also favored by hypoxia, as occurs in the development of the premetastatic niche [23,24]. Exosomal uptake is performed through various mechanisms, including phagocytosis, membrane fusion, lipid-raft-mediated endocytosis, clathrin-mediated endocytosis, macropinocytosis, and caveolin-mediated endocytosis [25] – Figure 2. The release and uptake of exosomes are accelerated in acidic pH environments, as can be found in the primary tumor microenvironment [26]. 

## 3. The Role of Small Extracellular Vesicles at the Primary Tumor site

Hypoxia is responsible for tumoral progression, cancer metastasis, and therapy resistance and is present in all solid tumors with volumes over 1 cm^3^ as a consequence of insufficient blood supply caused by aberrant tumoral microcirculation [27]. For solid tumors to constantly develop, neoangiogenesis is a vital process in response to hypoxia. VEGF (vascular endothelial growth factor) and PlGF (placental growth factor) are two soluble factors released by solid tumors that are responsible for vascular remodeling. Besides these two factors, small EVs have a proangiogenic effect upon vessels that surround the primary tumor [7,28]. In hypoxic conditions, multiple myeloma cells were shown to release significant numbers of small EVs loaded with miR-135b, which promotes local neoangiogenesis by enhancing endothelial tube formation [29].

Tumors are made of many genetically and phenotypically different cell subclones. It seems that, within the primary tumor microenvironment, different subclones can transfer oncogenic features into neighboring cells using small EVs [30]. For example, small EVs released by metastatic melanoma cells can modify the phenotypes of neighboring nonmetastatic melanoma cells, transforming them into metastatic cells [31]. 

Small EVs released by the primary tumor cells contain molecular constituents that induce cellular immune dysfunction, reducing the antitumoral immune response either by altering the function of immune cells or by modifying the nonimmune stromal cell phenotype [32]. T-cell immunity can be suppressed by the small EVs released from the primary tumor through a mechanism that results in cytotoxic T-cell apoptosis leading to a tumor-promoting immune cell phenotype [33].

## 4. The Role of Small Extracellular Vesicles in Cerebral Premetastatic Niche Development 

The concept of the premetastatic niche was first proposed and demonstrated by David Lyden [34]. A premetastatic niche describes the formation of a specific microenvironment in a distant organ that is devoid of cancer cells, and this process is induced by the primary tumor, which releases various tumor factors and small EVs that travel to secondary organs where they remodel the extracellular matrix, modify the vascular permeability, and alter the immune system by fusing with the resident cells and transferring their cargo of proteins, various metabolites, or genetic material. These premetastatic niches fulfill the perfect conditions for hosting the upcoming metastatic cells for survival and outgrowth [35,36,37,38,39]. Fong et al. [40] demonstrated that small EVs loaded with miR122 released from breast cancer cells reduce the glucose uptake of normal cells in secondary organs to preserve the nutrients for the incoming metastatic cells. Surgical intervention is another demonstrated factor that contributes to the formation of premetastatic niches. Surgery leads to local hypoxia and local injury, which are known to promote metastasis and tumor cell shedding into the bloodstream, along with a surge of inflammatory cells that promote premetastatic niche formation [41,42,43]. 

In the clinical stage of cerebral metastases, small EVs released from secondary sites seem to be as important as those released by the primary cancer cells; for example, the small EVs released by normal astrocytes decrease the expression of the phosphatase and tensin homolog gene (PTEN), which is an important tumor suppressor in brain metastases. Zhang et al. [44] showed that tumor cells with normal PTEN expression lose their PTEN expression after spreading into the brain. The PTEN levels remained normal after the dissemination of tumor cells into other organs. Furthermore, brain metastatic cells with decreased PTEN expression regain their PTEN levels after removal from the brain microenvironment. This mechanism is explained by the fact that astrocytic cells release small EVs containing PTEN-targeting miR-19a that is transferred to brain metastatic tumor cells, which then lose their PTEN expression. Moreover, the PTEN loss of the brain metastatic tumor cells increases the secretion of the CCL2 chemokine and the recruitment of IBA1-expressing myeloid cells that further enhance the brain metastatic process by intensifying the proliferation and reducing the apoptosis of metastatic tumor cells.

The metastatic process, also known as the metastatic cascade, is composed of the following steps that tumor cells must undergo to successfully disseminate into secondary organs: local invasion, immune response survival, intravasation inside the bloodstream, and extravasation in the secondary organs. Small EVs enhance systemic invasion and cancer cell progression in the metastatic cascade [45]. 

For specific brain metastases to develop, tumor cells must pass through the tight junctions of the blood–brain barrier (BBB) and survive inside the brain parenchyma. The majority of cancer cells die instantly after crossing the BBB, so, even now, the underlying mechanisms of the cerebral metastatic process remain to be clarified [46]. Despite being a barrier against cancer cells, the BBB has been shown to aid tumor development. After transmigration, tumor cells remain in close contact with the basolateral side of the microvascular endothelium of the brain, proliferating in this location; when the tumor cells are displaced or removed from this position, the cancerous cells gradually die [47]. 

In the metastasis of lung cancer cells towards the brain parenchyma, the BBB permeability markedly increases, allowing circulating cancer cells to penetrate the brain parenchyma; however, these mechanisms are not fully understood. Wu et al. [48] showed, with endothelial monolayers and mouse models, that small EVs enriched in lnc-MMP2-2 (long noncoding-matrix metalloproteinase2-2) can weaken the BBB by destroying tight junctions, thus promoting the metastasis of non-small-cell lung cancer; moreover, he concluded that lnc-MMP2-2 knockdown significantly decreases the in vivo brain metastasis of NSCLC. Lnc-MMP2-2 may become a diagnostic marker or a therapeutic target in the future. Both small-cell lung cancer (SCLC) and non-small-cell lung cancer (NSCLC) have a propensity for brain metastasis. The transendothelial migration of SCLC cells might be possible, because they might acquire various migration abilities by interacting with microvascular endothelial cells. Matrix metalloproteinase-9 expression significantly increases in brain metastases of lung adenocarcinoma, showing its important role in the transmigration of cancer cells across the BBB [49]. The increased expression of chemokine receptor 4 and CD44 on the surface of non-small-cell lung cancer cells is thought to contribute to brain metastasis [50,51]. In addition, SCLC cells release placental growth factors that weaken the BBB and facilitate brain metastasis [52]. 

Tominaga et al. [53] showed that small EVs containing microRNA181c released by metastatic breast cancer cells can promote brain metastasis, destroying the BBB by modulating the actin dynamics. Small EV microRNA181c expression is elevated in patients with brain metastases; however, in premetastatic patients, small EV microRNA181c expression has not yet been quantified.

When discussing the dissemination mechanism of cancerous cells that must traverse the BBB, we may wonder about the mechanism of adhesion of the tumoral cells to the endothelial cell layer of the brain, which may be a crucial step that precedes the transmigration of cancerous cells. Fazakas et al. [54] used atomic force microscopy to study the deadhesion strength of breast adenocarcinoma cells to the endothelial cell layer of the brain and concluded that the deadhesion strength of endothelial cells pretreated with small EVs released from breast adenocarcinoma cells was lower than that of nontreated endothelial cells. His results showed that this may not be a key step in breast adenocarcinoma metastasis development. 

Small EVs modulate vascular permeability and stimulate neoangiogenesis, two steps that are crucial in premetastatic niche establishment, resulting in the initial extravasation of tumor cells followed by metastatic cancer cell development in secondary organs. For example, MDA-MB-231 breast cancer metastatic cells release small EVs containing miR105 that help destroy the endothelial cell barrier by downregulating ZO-1 (zonula-occludens 1 protein) tight junctions, increasing the permeability of the endothelial barrier and permitting cancer cell metastasis into secondary organs [55,56]. Lu et al. [57], using an in vitro system, revealed that the BBB can be disrupted by small EV lncRNA GS1-600G8.5 derived from metastatic breast cancer cells that target tight junction proteins, promoting the brain metastasis of breast cancer cells.

Microglia are the immune cells found inside the brain that are activated by many pathological situations, such as infection or cancer of the neuroaxis. Microglia can have both tumor-suppressive (M1) and tumor-promoting (M2) roles. It has been shown that, preferentially inside the brain, XIST (X-inactive-specific transcript) works as a metastatic suppressor gene, as demonstrated by XIST downregulation promoting epithelial-mesenchymal transition (EMT), MSN/c-Met, and small EV miR-503 release, all of which leads to the development of metastatic traits in tumor stem cells and microenvironment reprogramming via small EV-mediated communication. The increased secretion of small EV miR-503 results in reprogramming of the microglia from M1 to M2, leading to immune suppression and the release of growth factors that promote tumor cell survival and migration in patients with breast cancer, thus promoting brain metastasis. Serum small EV miR-503 may be used in the future as a biomarker to assess the risk of developing brain metastases in patients with breast cancer [58].

## 5. Exosomal Oncoproteins Involved in the Cerebral Metastatic Process

Exosomes have various transmembrane, intraluminal, or surface-attached proteins. Tetraspanins are integral exosomal membrane proteins that mediate the exosomal inclusion of various proteins, such as integrins, which play a crucial role in the development of the premetastatic niche and organotropism of lung and liver metastases. Curiously, small EVs released by brain metastases pack very small amounts of integrins, suggesting that other exosomal molecules may support brain metastasis [38,59]. However, higher levels of circulating small EVs enriched in integrin β3 are associated with worse survival in patients following whole-brain radiotherapy for lung cancer brain metastases [60].

Only a few organs host deposits of metastatic cells, although cancerous cells can be found in the vascular beds of many organs, further emphasizing the role of small EVs as key factors in the metastatic process. Rahman et al. [15] showed that small EVs released from highly metastatic lung cancer cells or those from late-stage lung cancer serum induce EMT, a process resulting in the migration, invasion, and metastasis of cancer cells, and upregulate vimentin, concluding that small EVs may drive the metastatic spread of lung cancer. Vimentin is an intermediate filament protein transported by small EVs and released by the cancer cells whose expression increases in the metastatic stage of lung cancer, resulting in a poor prognosis for the patient. 

Another protein carried by small EVs and released from brain metastatic lung and breast cancer cells is the cell migration-inducing and hyaluronan-binding protein (CEMIP or KIAA1199). This protein promotes brain metastasis by inducing a proinflammatory vascular niche in the early stages of metastatic colonization. Studies show that high CEMIP expression in primary lung or breast cancer cells is associated with shorter intervals between primary cancer and the metastatic stage, the rapid evolution of brain metastases, and a poor prognosis for the patient. CEMIP expression is very high in small EVs released by lung and breast brain metastases, although not in metastases located elsewhere or even in the primary lung or breast cancer cells. Additionally, CEMIP seems to be selectively packed in exosomes, because it occurs in ten times larger quantities in small EVs compared with brain metastatic cells [59]. 

S100A16, a protein in the S100 family, increases in SCLC cells located in the brain microvascular microenvironment. Small EVs derived from human brain microvascular endothelial cells (HBMEC) control S100A16 elevation. Experiments have shown that SCLC cells treated with small EVs released from HBMEC increase the S100A16 level, resulting in increased apoptosis resistance under stress and increased cell proliferation. Additionally, S100A16 upregulates PHB-1, a protein located in the inner mitochondrial membrane, preserving the mitochondrial membrane potential and, thus, improving the survival of metastatic SCLC cells in the cerebrum [46]. 

Puigdelloses et al. [61] showed that RNU6-1 expression is increased in glioblastoma patients and patients who present with cerebral metastases. RNU6-1 is a sncRNA (small noncoding RNA) involved in RNA processing and tumoral growth rate regulation.

The small EVs released from metastatic breast cancer cells increase the expression of miR210, annexin II, and integrinβ3. Small EVs released by metastatic lung cancer cells present an increased expression of TGF beta and IL-10. The drawback when using small EVs as biomarkers is that all the cells release small EVs that can interfere with the small EVs released by tumoral cells. One way to solve this situation is by using cancer-specific markers to separate tumor-secreted small EVs from the small EVs released by normal cells [21]. A summary of all the exosomal oncoproteins and exosomal cargoes presented in this review can be seen in Table 1 below. 

## 6. Exosomes: Future Directions 

Small EVs may be used as a minimally invasive way to track brain metastases or other primary brain tumors and assess the response to various treatment methods, such as surgical treatment and chemo- or radiotherapy, by obtaining exosomal profiles before and after treatment. The use of small EVs as liquid biopsies could be beneficial for situations in which imaging findings do not reflect the present tumor biology, as when a differential diagnosis must be made between tumor recurrence and radiation necrosis or tumor pseudo-progression, which results in an increased contrast enhancement on imaging. Liquid biopsies may also be used when a patient is treated with antiangiogenic agents that reduce the contrast enhancement by decreasing the permeability of the tumor vascularity without actually reducing the tumoral size. Using small EVs as liquid biopsies will significantly decrease the risks associated with classic biopsies [62,63]. 

Chemotherapy is known to be inefficient in brain metastases, because drugs have to traverse the BBB to be efficient. In the future, small EVs will be used as carriers for therapeutic agents across the BBB. Liu et al. [64] used CXCR4/TRAIL-enriched small EVs obtained from mesenchymal stem cells as cooperative agents with carboplatin against the cerebral metastases of breast cancer in vivo, improving the efficacy of chemotherapy. Yang et al. [65] delivered siRNA across the blood–brain barrier in zebrafish with natural endothelial cell-derived small EVs as nanocarriers. Yang et al. [66] showed that, through cellular nanoporation, small EVs may be used as universal nucleic acid carriers in applications that require transcriptional manipulation. Small EVs as drug carriers were also demonstrated by Lai et al. [67], who showed that subarachnoid hemorrhage cerebral edema in mice can be alleviated with small EVs loaded with miR-193b-3p that cross the BBB and deliver miR-193b-3p to the affected regions. 

Better understanding the process of tumor exosomal biogenesis may lead to decreased metastasis through the development of new techniques to control tumor exosomal production. Goals for the future include the development of new ways to identify the noncancerous premetastatic sites developed in secondary tumor-free organs early by combining the advances in serum biomarker identification, imaging, and the continuously accumulating knowledge about the premetastatic niche development. The early identification of premetastatic niches could allow them to serve as prognosis biomarkers and therapeutic targets to prevent metastasis development [38,68].

Recently, cancer stem cell (CSC) studying has gained much interest. It is known that CSCs contribute to tumorigenesis, metastasis, and therapeutical resistance, because these cells have the ability to self-renew and to differentiate into various cell types. CSC present chemo and radio resistance, attributable to EMT, DNA damage checkpoint activation, the upregulation of CSC markers, and the signaling pathways. This makes them extremely sought-after targets in the battle against resistance to treatment and tumoral recurrences. CSCs have been identified in solid tumors such as brain gliomas, breast, ovarian, colorectal, prostate, and pancreatic cancer, as well as in hematologic malignant diseases such as leukemia. Small EVs released by the CSC represent a new area of interest, because they transmit information between CSC and non-CSC, resulting in the activation of the CSC and modification of the surrounding microenvironment, finally contributing to cancer progression. Brain tumor CSCs express the exosomal markers TSG 101 (tumor susceptibility gene) and flotillin 1 which can suppress T-cell activity by the upregulation of tenascin-C. New ways of targeting these small EVs may finally lead to elimination of this CSC, resulting in curing the patient. This subject still represents a novelty, and further studying is needed [69,70,71].

## 7. Conclusions 

Small EVs represent an important target that may be used for early cancer diagnosis, prognosis prediction, cancer treatment, or to track the responses to various treatment methods. Their use in the diagnosis of central nervous system metastasis as liquid biopsies derived from blood or CSF shows promise, especially in deep-seated lesions where stereotactic biopsies may be dangerous or in differential diagnosis with primary gliomas. Small EVs can be used not only for diagnostic purposes but also for molecular tumor profiling.

## Figures and Tables

**Table 1:** The importance of the exosomal cargo in the brain metastatic process.

Exosomal Origin	Exosomal Cargo	Role in Brain Metastatic Process	References
Multiple myeloma	miR-135b	promotes neoangiogenesis in the primary tumor	Umezu et al. [29]
Breast cancer	miR122	↓ glucose uptake of normal brain cells	Fong et al. [40]
Normal astrocytic cells	PTEN targeting miR19a	↓ PTEN expression in brain metastatic tumor cells	Zhang et al. [44]
Non-small cell lung cancer	lnc-MMP2-2	destroys the tight junctions of the BBB	Wu et al. [48]
Breast cancer	microRNA 181c	destroys the BBB by modulating the actin dynamics	Tominaga et al. [53]
Breast cancer	miR105	destroys the endothelial cell barrier by down-regulating ZO-1 tight junctions	Lokody et al. [55]
Breast cancer	lncRNA GS1-600G8.5	disrupts the BBB by targeting the tight junction proteins	Lu et al. [57]
Breast cancer	miR-503	induces the release of tumoral growth factors and microglial reprograming leading to immune suppression	Xing et al. [58]
Lung cancer	Vimentin	vimentin expression ↑ in the metastatic stage and induces epithelial–mesenchymal transition	Rahman et al. [15]
Lung and breast cancer	CEMIP	induces a proinflammatory vascular niche, promoting metastasis	Rodrigues et al. [59]
Lung cancer SCLC	S100A16	improves the survival of SCLC metastatic cells inside the cerebrum	Xu et al. [46]
Lung cancer, breast cancer, colorectal cancer, melanoma, pancreatic cancer, gastroesophageal cancer, bladder cancer	RNU6-1	regulates tumoral growth rate	Puigdelloses et al. [61]
